# Real-world safety of nivolumab in patients with malignant pleural mesothelioma in Japan: post-marketing surveillance study

**DOI:** 10.1093/jjco/hyae119

**Published:** 2024-09-03

**Authors:** Nobukazu Fujimoto, Ayumi Akamatsu, Chikara Honda, Miki Aoki, Yuichiro Ohe

**Affiliations:** Department of Medical Oncology, Okayama Rosai Hospital, Okayama, Japan; Pharmacovigilance Division, Ono Pharmaceutical, Osaka, Japan; Pharmacovigilance Division, Ono Pharmaceutical, Osaka, Japan; Pharmacovigilance Division, Ono Pharmaceutical, Osaka, Japan; Department of Thoracic Oncology, National Cancer Center Hospital, Tokyo, Japan

**Keywords:** malignant pleural mesothelioma, nivolumab, Japan, post-marketing surveillance, safety

## Abstract

**Objective:**

This post-marketing surveillance (PMS) was conducted to evaluate the incidence of adverse events with nivolumab in patients with unresectable, advanced or recurrent malignant pleural mesothelioma (MPM) that had progressed after first-line chemotherapy and to identify factors that potentially affected its safety in real-world clinical practice.

**Methods:**

Patients who had not received nivolumab previously were registered between November 2018 and February 2021. Nivolumab was given intravenously 240 mg every 2 weeks or 480 mg every 4 weeks. Patients were followed up for 6 months after treatment initiation. Information on patient characteristics, treatment status, and adverse events was collected.

**Results:**

This PMS enrolled 124 patients, involving 48 sites across Japan. At 6 months, nivolumab therapy was ongoing in 35.5% of patients (44/124) and had been discontinued in 64.5% (80/124). The overall incidence of treatment-related adverse events (TRAEs) was 40.3%; the incidence of Grade 3 or higher TRAEs was 12.9%. The pattern of TRAEs based on System Organ Class categories was generally consistent with those seen in the Japanese phase II MERIT study. The most common Grade 3 or higher TRAEs were interstitial lung disease (2.4%), lung disorder, and diarrhea (each 1.6%). The incidence of TRAEs was significantly higher in inpatients or patients who had good PS, high bodyweight, high body mass index, or autoimmune diseases than in those without these characteristics.

**Conclusion:**

The post-marketing incidence of TRAEs with nivolumab in patients with MPM has been evaluated, and no new safety signals were identified compared to the phase II clinical trial in Japan.

## Teaser

In this post-marketing surveillance study of nivolumab in patients with malignant pleural mesothelioma in Japan, no new safety signals were identified compared with phase II clinical trial data.

## Introduction

Malignant pleural mesothelioma (MPM), the most common form of malignant mesothelioma, is a disease with a poor prognosis that is caused primarily by asbestos exposure [1]. In Japan, the number of deaths from MPM in 2000 was 710 but this has been increasing year by year to 1605 in 2020, and the peak is expected to occur in 2027 [1,2].

Traditionally, recommended first-line systemic therapeutic options for MPM include cisplatin plus pemetrexed chemotherapy (with or without bevacizumab). Currently, nivolumab (a human monoclonal antibody against human programmed cell death [PD]-1) plus ipilimumab (a cytotoxic T lymphocyte antigen 4 inhibitor) has been added to first-line treatment options for MPM [3–5]. Treatment options for second-line therapy are limited; Japanese clinical practice guidelines for MPM recommend nivolumab monotherapy [3], while NCCN and ESMO guidelines recommend nivolumab plus ipilimumab, or nivolumab monotherapy, as immunotherapy options [4,5].

Nivolumab has demonstrated efficacy in the treatment of MPM, as monotherapy in the second-line setting or in combination with ipilimumab as first-line therapy [6–10]. In Japan, nivolumab was approved as monotherapy in August 2018 for the treatment of ‘unresectable, advanced or recurrent MPM that has progressed after first-line chemotherapy’, based on the results of a single arm, Japanese phase II clinical study (MERIT study/ONO-4538-41) [10].

Since the MERIT study had a limited sample size of 34, the impact on safety in real-world clinical practice in Japan remains unclear. In addition, globally, there is extremely limited real-world data regarding immune checkpoint inhibitor (ICI) therapy for MPM after first-line treatment [11]. Therefore, we conducted post-marketing surveillance (PMS) to evaluate the incidence of adverse events with nivolumab in patients with unresectable, advanced or recurrent MPM that had progressed after cancer chemotherapy, and to examine the factors that potentially affected safety in these patients in the real-world setting.

## Methods

### Study design

This PMS was conducted prospectively at 48 sites in Japan. The overall surveillance period was from 1 November 2018 to 31 August 2022, with the patient registration period lasting from 1 November 2018 to 28 February 2021. The duration of observation for each patient was 6 months from the first dose of nivolumab. This duration was chosen because in the MERIT clinical study, 94.7% of adverse events occurred within the first 6 months of initiating treatment with no apparent trend of increasing adverse events over time. Patient outcomes and adverse events were collected until the end of the 6-month observation period wherever possible, including for patients who completed or discontinued nivolumab use within that period. Data were collected using case report forms.

A written contract for this PMS was concluded between each participating site and the sponsor company. The PMS was registered with the Japan Registry of Clinical Trials (jRCT2011230029) and was conducted in accordance with the Ministerial Ordinance on Good Post-marketing Study Practice.

### Patients

Patients were eligible for surveillance if they had unresectable, advanced or recurrent MPM that had progressed after first-line chemotherapy and had not received nivolumab previously. In September 2020, the labeled dosage and regimen for nivolumab was amended to include 480 mg every 4 weeks in addition to the already included condition of 240 mg every 2 weeks. Therefore, the study population of this PMS included patients who received nivolumab intravenously 240 mg every 2 weeks and/or 480 mg every 4 weeks.

### Assessments

Baseline characteristics of the patients were collected, including sex, age, Eastern Cooperative Oncology Group (ECOG) Performance Status (PS), smoking history, medical history, asbestos exposure history, primary tumor site, presence/absence of pleural effusion, prior therapies, and inpatient/outpatient status. Nivolumab dosing details, including therapy dates, numbers of doses administered, and reason for discontinuation, were collected. Concomitant medication/therapy was also recorded. Information on adverse events was collected after the start of nivolumab use until the end of the observation period. The following information was reported: terms of adverse events, dates of onset, National Cancer Institute Common Terminology Criteria for Adverse Events grades, seriousness, causal relationship to nivolumab, other suspected causative factors, actions taken with respect to nivolumab treatment, treatment for adverse events, outcomes of adverse events, and laboratory test data related to adverse events. Treatment-related adverse events (TRAEs) were defined as adverse events for which a causal relationship to nivolumab could not be denied. Adverse events were classified using the Medical Dictionary for Regulatory Activities/Japanese version 25.0 and indicated by system organ class and preferred term. Patient outcome status (alive, dead, and unknown) at 6 months after starting nivolumab was also recorded.

### Statistical analysis

The sample size was set at 100 patients eligible for safety analysis, to enable a comparison with MERIT study data. In the MERIT study, adverse events with the lowest incidence occurred in 2.9% of patients (1/34). With a sample size of 100, the power to detect a frequency of 2.9% (probability of detecting an event occurring at the same rate observed in one patient in the MERIT study) would be 94.7%, which allows assessment of adverse events that were known to have occurred in the MERIT study. The safety analysis population included patients who received at least one dose of nivolumab and for whom data on the presence/absence of adverse events were available. Descriptive statistics were reported.

The main analysis focused on TRAEs. To identify factors that may affect safety, subgroup analysis of the incidence of TRAEs was performed, and the incidence in different subgroups was compared using Fisher’s exact test, Wilcoxon rank sum test, or Chi-square test. Data were analyzed using SAS version 9.4 (SAS Institute, Cary, NC, USA). All analyses in this PMS were pre-planned.

## Results

### Patients

**Figure 1:**
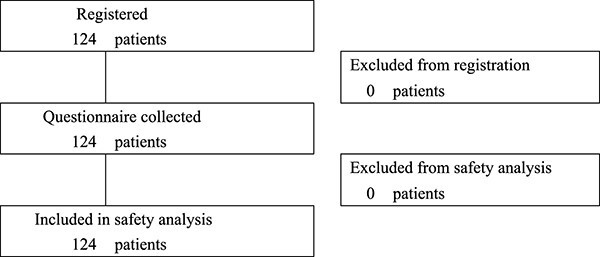
Disposition of patients.

A total of 124 patients were registered from 48 sites between 1 November 2018 and 28 February 2021. All 124 patients were included in the safety analysis (Fig. 1). The patients’ background characteristics are summarized in Table 1 and Table 2. Male patients accounted for 83.9% of the study population (104/124). The median age of patients at registration was 71.0 years (range 48–88), with those aged 75 years or older accounting for 34.7% of the study population (43/124). Most patients (91.9%) had an ECOG PS of 0 or 1; only 8.1% (10/124) had a PS of 2 or higher. The majority of patients (72.6%) were former smokers. Overall, 58.1% of patients (72/124) had a history of asbestos exposure, 25.8% (32/124) did not, and the status was not known for 16.1% (20/124). The median duration of asbestos exposure was 20.5 years (range 0–50) among those with a history. Histological types of MPM included epithelioid in 66.9% of patients (83/124), sarcomatoid in 14.5% (18/124), biphasic in 12.1% (15/124), and desmoplastic (fiber-forming) in 1.6% (2/124). More than half of patients (56.5%; 70/124) had a history of recent pleural effusion. Prior surgery for MPM had been performed in 23.4% of patients (29/124) and 10.5% of patients (13/124) had undergone prior radiation therapy. Almost half of all patients (59/124; 48.0%) had received pemetrexed + cisplatin before starting nivolumab.

**Table 1:** Patients’ baseline characteristics (N = 124)

Characteristic		Number of patients (%)	
Sex	Male	104	(83.9)
	Female	20	(16.1)
Age(Years)	<15	0	N/A
	15–<65	19	(15.3)
	65–<75	62	(50.0)
	≥75	43	(34.7)
	Mean ± SD	71.3 ± 7.5	
	Median	71.0	
	Min–Max	48–88	
ECOG PS	0	46	(37.1)
	1	68	(54.8)
	2	8	(6.5)
	3	2	(1.6)
	4	0	N/A
Smoking status	Never	27	(21.8)
	Current	6	(4.8)
	Former	90	(72.6)
	Unknown	1	(0.8)
Medical history: lungs	No	112	(90.3)
	Yes	12	(9.7)
Medical history: liver	No	112	(90.3)
	Yes	12	(9.7)
Medical history: kidney	No	112	(90.3)
	Yes	12	(9.7)
Medical history: thyroid	No	120	(96.8)
	Yes	4	(3.2)
Medical history: autoimmune disease	No	119	(96.0)
	Yes	5	(4.0)

ECOG PS: Eastern Cooperative Oncology Group performance status; Max: maximum; Min: minimum; SD: standard deviation.

N/A: not applicable.

**Table 2:** Mesothelioma and asbestos exposure history

Characteristic		Number of patients (%)	
History of asbestos exposure	No	32	(25.8)
	Yes	72	(58.1)
	Not clear	20	(16.1)
Years of asbestos exposure	<10 years	7	(9.7)
	10–<20 years	4	(5.6)
	20–<30 years	7	(9.7)
	30–<40 years	3	(4.2)
	≥40 years	5	(6.9)
	Not clear	46	(63.9)
	Number of patients	26	
	Mean ± SD	21.6 ± 15.4	
	Median	20.5	
	Min–Max	0–50	
Primary tumor location	Right pleura	67	(54.0)
	Left pleura	52	(41.9)
	Both	2	(1.6)
	Others	3	(2.4)
Histological type	Epithelioid	83	(66.9)
	Sarcomatoid	18	(14.5)
	Biphasic	15	(12.1)
	Desmoplastic (fiber-forming)	2	(1.6)
	Other	0	N/A
	Not clear	6	(4.8)
Recent pleural effusion before starting nivolumab^a^	No	54	(43.5)
	Yes	70	(56.5)
	Not recorded	0	N/A
Prior surgery	No	95	(76.6)
	Yes	29	(23.4)
Prior radiation therapy	No	111	(89.5)
	Yes	13	(10.5)
Recent chemotherapy before starting nivolumab	Pemetrexed + cisplatin	59	(48.0)
	Pemetrexed + carboplatin	36	(29.3)
	Pemetrexed	15	(12.2)
	Other	13	(10.6)

Max: maximum; Min: minimum; SD: standard deviation. N/A: not applicable.

^a^At last assessment before baseline.

### Treatment

In September 2020, which is during the study period, the labeled dosage and regimen for nivolumab was expanded to include 480 mg every 4 weeks in addition to the already used dosage of 240 mg every 2 weeks. Among the participants of this PMS, 110 patients (88.7%) received only doses of 240 mg during the observation period, while nine patients (7.3%) switched to the 480 mg dosage during the observation period and three patients (2.4%) received only doses of 480 mg. The median number of nivolumab doses administered was 7.5 (range 1–14) in the overall population. By age, the median number of nivolumab doses was 7.0 (range 1–14) for patients aged younger than 75 years and 9.0 (range 1–14) for patients aged 75 years or older. By ECOG PS scores, the median number of nivolumab doses for patients with an ECOG PS of 1 and below was 8.0 (range 1–14), while the median was 1.0 (range 1–12) and 3.0 (range 1–5) for patients with an ECOG PS of 2 and 3, respectively.

At 6 months after the initiation of treatment (the end of the observation period), nivolumab therapy was ongoing in 35.5% of patients (44/124) and had been discontinued in 64.5% (80/124). The most common reasons for treatment discontinuation were progression of the underlying disease including death (28.2%; 35/124), occurrence of an adverse event (21.8%; 27/124) and because the investigator judged that the effect of the drug could not be expected (18.5%; 23/124). No patients underwent surgical therapy or received concomitant immunotherapy during the observation period; three patients (2.4%) received radiation therapy.

### Safety and tolerability

The overall incidence of adverse events was 46.8% (58/124), and the incidence of serious adverse events was 31.5% (39/124). The overall incidence of TRAEs was 40.3% (50/124), and the incidence of Grade 3 or higher TRAEs was 12.9%. The most common TRAEs by System Organ Class (SOC) were ‘Respiratory, thoracic, and mediastinal disorders’, ‘Skin and subcutaneous tissue disorders’, ‘General/systemic disorders and administration site conditions’, and ‘Gastrointestinal disorders’. The incidence of TRAEs, based on SOC categories, showed no apparent differences compared with the trends seen in the MERIT study at the time of nivolumab approval in Japan (August 2018) (Table 3 and See online supplementary material for a colour version of Suppl. Table S1). For most of the TRAE categories by SOC, the incidence in the current PMS was lower than, or similar to, that in the MERIT study. However, the incidence of TRAEs for the SOC ‘Respiratory, thoracic, and mediastinal disorders’ was higher in this PMS (9.7%; 12/124) than in the MERIT study (5.9%; 2/34). Among the 12 patients who developed ‘Respiratory, thoracic, and mediastinal disorders’ in this PMS, at the PT level, 6 patients had interstitial lung disease (ILD; all of which resulted in discontinuation of nivolumab), 4 patients had lung disorder (resulting in nivolumab interruption or discontinuation) and 2 patients had pneumonitis (resulting in nivolumab continuation or interruption) (See online supplementary material for a colour version of Suppl. Table S1 and Suppl. Table S2). ILD was observed in 2 of 5 cases with a history of interstitial pneumonia. The most common individual TRAEs in this PMS were rash (5.6%; 7/124), ILD (4.8%; 6/124), fever (4.8%; 6/124), hypothyroidism (4.0%; 5/124), lung disorder (3.2%; 4/124), and diarrhea (3.2%; 4/124).

**Table 3:** Incidence of treatment-related adverse events (TRAEs) according to system organ class (SOC) in this PMS and in the MERIT study

Type of adverse reaction (SOC) n (%)	MERIT study^a^ (N = 34)	PMS (N = 124)
Total TRAEs	23 (67.6)	50 (40.3)
Infectious and parasitic diseases	1 (2.9)	3 (2.4)
Blood and lymphatic system disorders	0 (−)	1 (0.8)
Endocrine disorders	2 (5.9)	6 (4.8)
Metabolic and nutritional disorders	4 (11.8)	1 (0.8)
Nervous system disorders	2 (5.9)	3 (2.4)
Eye disorders	1 (2.9)	1 (0.8)
Vascular disorders	0 (−)	1 (0.8)
Respiratory, thoracic, and mediastinal disorders	2 (5.9)	12 (9.7)
Gastrointestinal disorders	9 (26.5)	9 (7.3)
Hepatobiliary disorders	0 (−)	2 (1.6)
Skin and subcutaneous tissue disorders	8 (23.5)	11 (8.9)
Musculoskeletal and connective tissue disorders	4 (11.8)	2 (1.6)
Renal and urinary tract disorders	0 (−)	2 (1.6)
Reproductive system and breast disorders	1 (2.9)	0 (−)
General/systemic disorders and administration site conditions	8 (23.5)	10 (8.1)
Clinical examination	9 (26.5)	3 (2.4)
Injury, poisoning, and treatment complications	0 (−)	1 (0.8)

TRAEs were coded using MedDRA/J, Ver. 25.0, and are presented by System Organ Class (SOC).

^a^TRAEs reported among participants of the Japanese phase 2 clinical study (MERIT study) at the time of approval of nivolumab in Japan (August 2018). Data for the MERIT study up to March 2018 and after 3 years of follow up (November 2019) have been published previously [10,12].

Grade 3 or higher TRAEs occurred in 12.9% of patients (16/124). The most common Grade 3 or higher TRAEs (reported in two or more patients) were ILD (2.4%; 3/124), lung disorder, diarrhea, and death (each in 1.6%; 2/124). Treatment-related deaths occurred in two patients; however, detailed information on the cause of deaths was unknown according to a report from investigators and, therefore, they were regarded as treatment-related deaths. Among the 124 patients eligible for the safety analysis, 43 (34.7%) were elderly (aged 75 years or older). The incidence of Grade 3 or higher TRAEs in patients aged 75 years or older was 7.0% (3/43), comprising immune thrombocytopenia, adrenal insufficiency, and ILD, each occurred in 1 patient (2.3%). Among the 124 patients eligible for safety analysis, 10 (8.1%) had an ECOG PS score of 2 or above. No TRAEs occurred in patients with an ECOG PS score of 2 or above.

The time to onset of TRAEs is summarized in Table 4. The median time to onset ranged from 18.5 days (range 1–181) for ‘General disorders and administration site conditions’ to 98.5 days (range 57–176) for ‘Endocrine disorders’. The median time to onset of TRAEs within the most common SOC, ‘Respiratory, thoracic, and mediastinal disorders’, was 34.5 days (range 15–176).

**Table 4:** Time to onset of treatment-related adverse events (TRAEs)

Type of adverse reaction (SOC)	No. of patients	Time to onset (days)	
		Median	Range
Endocrine disorders	6	98.5	57–176
Respiratory, thoracic, and mediastinal disorders	12	34.5	15–176
Gastrointestinal disorders	9	55.5	2–179
Skin and subcutaneous tissue disorders	11	46.0	1–129
General disorders and administration site conditions	10	18.5	1–181

TRAEs were coded using MedDRA/J, Ver. 25.0, and are presented by System Organ Class (SOC).

When the same adverse reaction occurred more than once in the same patient, the adverse reaction was counted only once using its first episode. The table shows data for adverse reaction SOCs with an incidence of 5% (six patients) or more.

### Risk factors for TRAEs among patient background factors

The incidence of TRAEs was statistically significantly higher among inpatients compared with outpatients (49.4% [40/81] versus 23.3% [10/43], *P* = 0.007), and those who had a good PS (43.9% [50/114] with PS score 0–1 versus 0% [0/10] for PS score 2–4, *P* = 0.007), higher bodyweight (*P* = 0.024), a higher BMI (*P* = 0.050), or autoimmune diseases (100% [5/5] versus 37.8% [45/119], *P* = 0.009) (Table 5).

**Table 5:** Incidence of treatment-related adverse events (TRAEs) by patient background factor: Factors for which there was a significant difference in the incidence of adverse drug reactions between subgroups

Patient background factor		Number of patients (%)		Incidence of TRAEs			
				Number of patients	Incidence rate (%)	95% CI for incidence rate^a^	*P* value
Inpatient/Outpatient	Inpatient	81	(65.3)	40	(49.4)	[38.1, 60.7]	***P* = .007** ^b^
	Outpatient	43	(34.7)	10	(23.3)	[11.8, 38.6]	
ECOG PS	0	46	(37.1)	22	(47.8)	[32.9, 63.1]	***P* = .042** ^c^
	1	68	(54.8)	28	(41.2)	[29.4, 53.8]	
	2	8	(6.5)	0	N/A	[0.00, 36.9]	
	3	2	(1.6)	0	N/A	[0.00, 84.2]	
	4	0	N/A	0	N/A	N/A	
ECOG PS	0–1	114	(91.9)	50	(43.9)	[34.6, 53.5]	***P* = .007** ^c^
	2–4	10	(8.1)	0	N/A	[0.00, 30.9]	
Body weight (kg)	<50	24	(19.4)	7	(29.2)	[12.6, 51.1]	***P* = .024** ^c^
	50–<60	48	(38.7)	16	(33.3)	[20.4, 48.4]	
	60–<70	36	(29.0)	19	(52.8)	[35.5, 69.6]	
	70–<80	11	(8.9)	5	(45.5)	[16.8, 76.6]	
	≥80	4	(3.2)	3	(75.0)	[19.4, 99.4]	
	Not clear	1	(0.8)	0	N/A	N/A	
	Number of patients	123					
	Mean ± SD	58.2 ± 10.3					
	Median	57.0					
	Min–Max	40.0–85.5					
Body mass index (kg/m^2^)	<18.5	14	(11.3)	3	(21.4)	[4.7, 50.8]	***P* = .050** ^c^
	≥18.5–<25	85	(68.5)	34	(40.0)	[29.5, 51.2]	
	≥25	24	(19.4)	13	(54.2)	[32.8, 74.5]	
	Not clear	1	(0.8)	0	N/A	N/A	
	Number of patients	123					
	Mean ± SD	22.1 ± 3.3					
	Median	21.8					
	Min–Max	15.1–32.4					
Medical history: autoimmune disease	No	119	(96.0)	45	(37.8)	[29.1, 47.2]	***P* = .009** ^b^
	Yes	5	(4.0)	5	(100.0)	[47.8, 100.0]	

CI: confidence interval; ECOG PS: Eastern Cooperative Oncology Group performance status; Max: maximum; Min: minimum; SD: standard deviation; N/A: not applicable

No significant differences were seen between subgroups based on other patient background factors, including: sex; age; pregnancy or breastfeeding status (women), partner’s pregnancy status (men); height; asbestos exposure history; medical history related to lungs, liver, kidneys, thyroid, ILD, emphysema/COPD, lung infection; primary tumor location, tumor tissue type; history of recent pleural effusion; past treatment for malignant pleural mesothelioma (surgery, radiation, drug therapy); concomitant therapies; and nivolumab dosage or number of doses.

^a^Calculated using the exact method.

^b^Fisher’s exact test.

^c^Wilcoxon rank sum test.

To determine whether inpatient/outpatient status or PS status were affected by exposure to nivolumab, the incidence of TRAEs was cross-tabulated according to each factor and the number of doses of nivolumab that were taken. There was no evidence of increased exposure to the drug among inpatients (See online supplementary material for a colour version of Suppl. Table S3). In addition, there was no statistically significant difference in the incidence of Grade 3 or higher events in inpatients (14.8%; 12/81) compared with outpatients (9.3%; 4/43). Cross-tabulation of TRAEs according to ECOG PS and number of doses indicated that patients with a good PS tended to receive more doses of nivolumab than those with a poor PS. In fact, when looking at patients who received nivolumab as few as 1 ~ 4 times, the incidence of TRAEs was 23.7% (27/114) in those with an ECOG PS of 0 or 1, and 80.0% (8/10) in those with an ECOG PS of 2 or higher (See online supplementary material for a colour version of Suppl. Table S4). Furthermore, cross-tabulation of TRAEs according to bodyweight (which also affects BMI) and ECOG PS indicated that heavier patients tended to have a good PS (See online supplementary material for a colour version of Suppl. Table S5). Finally, we observed no marked tendency for TRAEs related to autoimmune diseases to occur more frequently in patients with a history of autoimmune disease.

### Patient outcome

Among the 124 patients eligible for the safety analysis, death for any reason during the observation period was confirmed for 25 patients (20.2%) and survival was confirmed for 94 patients (75.8%); survival status was unknown for 5 patients (See online supplementary material for a colour version of Suppl. Table S6).

## Discussion

This PMS evaluated the safety profile of nivolumab in routine clinical practice in Japan. The PMS involved 124 patients with MPM that had progressed after first-line chemotherapy receiving nivolumab in Japan, which is a larger patient population than the 34 patients included in the Japanese phase II MERIT study. As a result, we observed an overall incidence of TRAEs of 40.3% and an incidence of 12.9% for Grade 3 or higher TRAEs, which were not higher than those observed in the MERIT study [10].

The most common TRAEs in the PMS were rash (5.6%), ILD (4.8%), fever (4.8%), hypothyroidism (4.0%), lung disorder (3.2%), and diarrhea (3.2%). In the MERIT study, the most common TRAEs reported by the time of nivolumab approval in Japan were rash, diarrhea, and increased lipase (each occurred in 11.8%; 4/34). The pattern of TRAEs by SOC in the current PMS was comparable to that of the MERIT study and no new safety concern was identified. The most notable difference was a higher incidence of TRAEs within the SOC categories of ‘Respiratory, thoracic, and mediastinal disorders’ in the PMS (9.7%) than in the MERIT study (5.9%). Among the SOC of ‘Respiratory, thoracic, and mediastinal disorders’, most TRAEs were ILD-related in both this PMS and the MERIT study. The MERIT study excluded patients ‘with a history or current ILD or pulmonary fibrosis diagnosed based on imaging or clinical findings,’ which may have affected the incidence of events under this SOC in the study. However, in the current PMS, among the 12 patients who experienced events within the SOC ‘Respiratory, thoracic, and mediastinal disorders’, only two had a medical history related to ILD. On this basis, the difference in the incidence of events under this SOC between the PMS and the MERIT study may be due to factors other than the medical history related to ILD. However, the small sample size of the MERIT study (n = 34) limits the interpretation of differences in the incidence of events.

As the current PMS was conducted in real-world clinical practice, the study population differed slightly from the MERIT study. Patients with a poorer ECOG PS (PS score of 2 or above) were excluded from the MERIT study, but they accounted for 8.1% (10/124) of the population in this PMS. Of note, no TRAEs were identified in patients with an ECOG PS score of 2 or above, but caution is warranted due to the small sample size. Among the 124 patients eligible for the safety analysis, 34.7% (43/124) were aged 75 years or older, and the incidence of Grade 3 or higher TRAEs was 7.0% (3/43), which was lower than the incidence of Grade 3 or higher TRAEs of 12.9% (16/124) in the overall population. The histological tumor types seen in patients included in this PMS were similar to those in the MERIT study [10]. Overall, we confirmed the safety of nivolumab in this real-world patient population, with no marked increase observed in patients with a characteristic that is associated with a poorer prognosis.

The safety profile of nivolumab in this PMS, particularly in terms of the Grade 3 or higher TRAEs, appears to be generally consistent with the profile from clinical trials involving patients with MPM performed in other countries [6,8,9]. In phase II studies in France and the Netherlands, involving patients with relapsed MPM after chemotherapy, the most common Grade 3 or higher TRAEs with nivolumab monotherapy were pneumonitis [8] and increased lipase [6]. In a phase III trial (CONFIRM) involving patients with relapsed pleural or peritoneal malignant mesothelioma in the UK, the most common Grade 3 or higher TRAEs with nivolumab monotherapy were diarrhea (3%) and infusion-related reaction (3%) [9], while in the current surveillance of patients with MPM the most common events were ILD, lung disorder, and diarrhea. While it is difficult to directly compare the results of clinical trials and PMS because of differences in study designs, we found no apparent differences in the safety signals between the current PMS and those previous studies with different racial demographics.

Nivolumab has been approved for various types of cancer, and the safety profile of nivolumab in this PMS does not appear to differ substantially from that reported in PMS for other cancer types, including renal cell carcinoma [13,14], malignant melanoma [15], metastatic head and neck cancer [16], and non–small cell lung cancer (NSCLC) [17]. With respect to lung malignancies, in the PMS of nivolumab for the second-line treatment of patients with NSCLC, the incidence of TRAEs was 47.1% (1697/3601 patients) and that of Grade 3 or higher TRAEs was 15.9% (573/3601 patients) [17], which were similar to the rates in the current PMS. The most common TRAEs (>4%) in NSCLC patients were ILD, hypothyroidism, and diarrhea [17], while in the current PMS, the most common events were rash, ILD, fever, and hypothyroidism.

The median time to onset of TRAEs with an incidence of 5% or more within the most common SOC, ‘Respiratory, thoracic and mediastinal disorders’ was 34.5 days, with the range of onset times (15–176 days) encompassing the early phase of nivolumab treatment through to late phase in the 6-month observation period. PMS studies of nivolumab for other indications have also reported that TRAEs with nivolumab may occur throughout the first 6 months, although the median time to onset for adverse events of special interest (such as ILD) tended to be 1–2 months [16,17].

Analysis of the incidence of TRAEs by patient background factor found significant differences in the incidence of TRAEs based on five factors: inpatient/outpatient status, PS, body weight, BMI, and history of autoimmune diseases. The reason for the increased incidence of TRAEs among inpatients is not clear because the duration of exposure to nivolumab did not increase between inpatients and outpatients. The incidence of TRAEs was higher in patients with a good ECOG PS. These patients tended to receive more doses of nivolumab than those with a poor ECOG PS, which may have affected the incidence of TRAEs. It is possible that more patients with a poor ECOG PS have died from the underlying disease before developing TRAEs, which could have affected the incidence of TRAEs according to the ECOG PS status (See online supplementary material for a colour version of Suppl. Table S5). PMS studies of nivolumab in patients with NSCLC or malignant melanoma have also found that the incidence of TRAEs was higher in patients with a good PS [15,17], with one also reporting that nivolumab exposure was higher in those with a good PS [15].

The incidence of TRAEs was higher in patients with higher bodyweight or BMI. Since a good ECOG PS was more common in patients with higher bodyweight than in patients with lower bodyweight, the ECOG PS status and its associated patient outcomes discussed above could have affected the incidence of TRAEs by bodyweight. Furthermore, given the general correlation between BMI and body weight, it was considered that the considerations related to body weight could also apply to BMI.

The subgroup of patients with a history of autoimmune disease was small (n = 5), making it difficult to draw conclusions about a possible relationship with TRAEs to nivolumab based on the current PMS. A PMS study of nivolumab in patients with advanced renal cell carcinoma found a significantly higher rate of TRAEs in patients with a history of autoimmune diseases (75.0% versus 48.8%, *P* < 0.05). Given the potential for a higher incidence of TRAEs in patients with a history of autoimmune diseases, careful monitoring is warranted. In summarizing the risk factors for TRAEs, it seems difficult to provide a clear clinical interpretation for all factors that showed a significantly higher incidence of TRAEs.

The limitations of this PMS include the absence of a control group, and the fact that follow-up was limited to 6 months, although most adverse events are expected to occur within this timeframe. Another limitation is that efficacy data were not collected in this PMS. The strengths include the large number of patients compared with the clinical trial performed in Japan, and that the results come from a cohort of patients treated in routine clinical practice. This enabled the safety profile of the drug to be evaluated based on real-world data.

## Conclusion

The PMS incidence of TRAEs with nivolumab in patients with MPM has been evaluated and no new safety signals were identified compared to the phase II MERIT clinical trial in Japan.

## Supplementary Material

Supplementary_data_hyae119

## Data Availability

The data underlying this article are available in the article and in its online supplementary material.
