# Supplementary material for: Real-world safety of nivolumab in patients with malignant pleural mesothelioma in Japan: post-marketing surveillance study
Source: Jpn J Clin Oncol. 2024 Sep 3;54(12):1321–8. doi: 10.1093/jjco/hyae119 (PMC11631123; doi:10.1093/jjco/hyae119)
Supplement: Supplementary_data_hyae119 [file supplementary_data_hyae119.docx]

**Supplementary Table S1. Incidence of treatment-related adverse events (TRAEs) according to preferred term (PT) in this PMS and in the MERIT study**

| **Type of adverse reaction (PT) n (%)** | | **MERIT study^a^ (N=34)** | **PMS (N=124)** |
| --- | --- | --- | --- |
| **Total TRAEs** | | 23 (67.6) | 50 (40.3) |
| **Infections and infestations** | | 1 (2.9) | 3 (2.4) |
|  | Laryngitis | 1 (2.9) | 0 (-) |
|  | Pneumonia | 0 (-) | 2 (1.6) |
|  | Septic shock | 0 (-) | 1 (0.8) |
| **Blood and lymphatic system disorders** | | 0 (-) | 1 (0.8) |
|  | Immune thrombocytopenia | 0 (-) | 1 (0.8) |
| **Endocrine disorders** | | 2 (5.9) | 6 (4.8) |
|  | Addison's disease | 0 (-) | 1 (0.8) |
|  | Adrenal insufficiency | 0 (-) | 1 (0.8) |
|  | Hyperthyroidism | 0 (-) | 1 (0.8) |
|  | Hypothyroidism | 2 (5.9) | 5 (4.0) |
| **Metabolism and nutrition disorders** | | 4 (11.8) | 1 (0.8) |
|  | Hyperkalaemia | 0 (-) | 1 (0.8) |
|  | Hyponatraemia | 1 (2.9) | 1 (0.8) |
|  | Hypophosphataemia | 1 (2.9) | 0 (-) |
|  | Decreased appetite | 2 (5.9) | 0 (-) |
|  | Type 1 diabetes mellitus | 1 (2.9) | 0 (-) |
| **Nervous system disorders** | | 2 (5.9) | 3 (2.4) |
|  | Dysgeusia | 1 (2.9) | 0 (-) |
|  | Headache | 0 (-) | 1 (0.8) |
|  | Neuropathy peripheral | 1 (2.9) | 2 (1.6) |
| **Eye disorders** | | 1 (2.9) | 1 (0.8) |
|  | Iritis | 0 (-) | 1 (0.8) |
|  | Retinal oedema | 1 (2.9) | 0 (-) |
| **Vascular disorders** | | 0 (-) | 1 (0.8) |
|  | Flushing | 0 (-) | 1 (0.8) |
| **Respiratory, thoracic and mediastinal disorders** | | 2 (5.9) | 12 (9.7) |
|  | Interstitial lung disease | 1 (2.9) | 6 (4.8) |
|  | Lung disorder | 0 (-) | 4 (3.2) |
|  | Pneumonitis | 1 (2.9) | 2 (1.6) |
| **Gastrointestinal disorders** | | 9 (26.5) | 9 (7.3) |
|  | Constipation | 1 (2.9) | 0 (-) |
|  | Diarrhoea | 4 (11.8) | 4 (3.2) |
|  | Gastrointestinal pain | 0 (-) | 1 (0.8) |
|  | Nausea | 2 (5.9) | 1 (0.8) |
|  | Stomatitis | 3 (8.8) | 1 (0.8) |
|  | Vomiting | 2 (5.9) | 0 (-) |
|  | Immune-mediated enterocolitis | 0 (-) | 2 (1.6) |
| **Hepatobiliary disorders** | | 0 (-) | 2 (1.6) |
|  | Cholangitis sclerosing | 0 (-) | 1 (0.8) |
|  | Liver disorder | 0 (-) | 1 (0.8) |
| **Skin and subcutaneous tissue disorders** | | 8 (23.5) | 11 (8.9) |
|  | Eczema | 1 (2.9) | 1 (0.8) |
|  | Pruritus | 0 (-) | 1 (0.8) |
|  | Psoriasis | 0 (-) | 1 (0.8) |
|  | Rash | 4 (11.8) | 7 (5.6) |
|  | Rash maculo-papular | 2 (5.9) | 0 (-) |
|  | Skin exfoliation | 1 (2.9) | 0 (-) |
|  | Urticaria | 1 (2.9) | 1 (0.8) |
| **Musculoskeletal and connective tissue disorders** | | 4 (11.8) | 2 (1.6) |
|  | Arthralgia | 3 (8.8) | 0 (-) |
|  | Arthritis | 1 (2.9) | 1 (0.8) |
|  | Rhabdomyolysis | 0 (-) | 1 (0.8) |
| **Renal and urinary disorders** | | 0 (-) | 2 (1.6) |
|  | Haematuria | 0 (-) | 1 (0.8) |
|  | Proteinuria | 0 (-) | 1 (0.8) |
|  | Acute kidney injury | 0 (-) | 1 (0.8) |
| **Reproductive system and breast disorders** | | 1 (2.9) | 0 (-) |
|  | Gynaecomastia | 1 (2.9) | 0 (-) |
| **General disorders and administration site conditions** | | 8 (23.5) | 10 (8.1) |
|  | Death | 0 (-) | 2 (1.6) |
|  | Fatigue | 2 (5.9) | 1 (0.8) |
|  | Malaise | 3 (8.8) | 1 (0.8) |
|  | Mucosal inflammation | 1 (2.9) | 0 (-) |
|  | Pyrexia | 2 (5.9) | 6 (4.8) |
| **Investigations** | | 9 (26.5) | 3 (2.4) |
|  | Alanine aminotransferase increased | 0 (-) | 1 (0.8) |
|  | Amylase increased | 3 (8.8) | 0 (-) |
|  | Aspartate aminotransferase increased | 0 (-) | 1 (0.8) |
|  | Blood lactate dehydrogenase increased | 0 (-) | 1 (0.8) |
|  | Blood thyroid stimulating hormone increased | 1 (2.9) | 0 (-) |
|  | Cortisol decreased | 0 (-) | 1 (0.8) |
|  | Gamma-glutamyltransferase increased | 1 (2.9) | 1 (0.8) |
|  | Lipase increased | 4 (11.8) | 0 (-) |
|  | Lymphocyte count decreased | 2 (5.9) | 0 (-) |
|  | Thyroid function test abnormal | 1 (2.9) | 0 (-) |
|  | Weight decreased | 1 (2.9) | 0 (-) |
|  | Tri-iodothyronine free abnormal | 1 (2.9) | 0 (-) |
| **Injury, poisoning and procedural complications** | | 0 (-) | 1 (0.8) |
|  | Infusion related reaction | 0 (-) | 1 (0.8) |

TRAEs were coded using MedDRA/J, Ver. 25.0, and are presented by preferred term (PT).

a TRAEs reported among participants of the Japanese phase 2 clinical study (MERIT study) at the time of approval of nivolumab in Japan (August 2018). Data for the MERIT study up to March 2018 and after 3 years of follow up (November 2019) have been published previously (10,17).

**Supplementary Table S2. Details of interstitial lung disease adverse reactions**

| **Case** | **Age category (years)** | **Sex** | **ECOG PS** | **Smoking status** | **Medical history: lungs** | **Dose** | **Type of adverse reaction (PT)** | **Time to onset of TRAEs (days)** | **Grade** | **Factors other than this drug** | **Action taken with this drug** | **Outcome** | **Details of treatment** |
| --- | --- | --- | --- | --- | --- | --- | --- | --- | --- | --- | --- | --- | --- |
| 1 | 60-<70 | Female | 1 | Former | No | Total dose 240 mg/body | Interstitial lung disease | 30 | 2 |  | Discontinuation | Recovering | Steroid therapy |
| 2 | 70-<80 | Male | 0 | Former | No | Total dose 240 mg/body | Lung disorder | 32 | 1 |  | Interruption | Recovering | Steroid therapy |
|  |  |  |  |  |  |  | Lung disorder | 50 | 3 | Primary disease | Discontinuation | Resolved with sequelae | Steroid therapy |
| 3 | 60-<70 | Male | 0 | Former | No | Total dose 240 mg/body | Pneumonitis | 176 | 2 |  | Continuing | Recovering | Steroid therapy |
| 4 | 70-<80 | Male | 1 | Former | No | Total dose 240 mg/body | Interstitial lung disease | 37 | 3 |  | Discontinuation | Recovering | Steroid therapy |
| 5 | 80- | Female | 1 | Former | No | Total dose 240 mg/body | Pneumonitis | 28 | 2 |  | Interruption | Not resolved | Steroid therapy |
| 6 | 50-<60 | Male | 1 | Former | Yes | Total dose 240 mg/body | Lung disorder | 15 | 2 |  | Interruption | Recovering | Steroid therapy |
| 7 | 80- | Male | 0 | Former | Yes | Total dose 240 mg/body | Lung disorder | 147 | 2 | Possibly chronic lower respiratory tract infection | Discontinuation | Recovering | Steroid therapy |
| 8 | 80- | Female | 0 | Current | No | Total dose 240 mg/body | Interstitial lung disease | 123 | 3 |  | Discontinuation | Resolved | Steroid therapy |
| 9 | 70-<80 | Male | 1 | Former | Yes | Total dose 240 mg/body | Lung disorder | 17 | 3 | Interstitial changes | Discontinuation | Recovering | Steroid therapy |
| 10 | 70-<80 | Female | 0 | Never | No | Total dose 240 mg/body | Interstitial lung disease | 15 | 2 |  | Discontinuation | Resolved | Steroid therapy |
| 11 | 70-<80 | Male | 1 | Former | No | Total dose 240 mg/body | Interstitial lung disease | 84 | 2 |  | Discontinuation | Resolved with sequelae | Steroid therapy Antibiotic treatment |
| 12 | 70-<80 | Male | 1 | Never | No | 240 mg/body to 480 mg/body | Interstitial lung disease | 39 | 3 | Primary disease | Discontinuation | Recovering | Steroid therapy Antibiotic treatment |

**Supplementary Table S3. Cross-tabulation of treatment-related adverse events (TRAEs; number of doses × inpatient/outpatient)**

| **Patient background factor** | | **Overall population** | | | | **Inpatient/Outpatient** | | | | | | | |
| --- | --- | --- | --- | --- | --- | --- | --- | --- | --- | --- | --- | --- | --- |
|  |  |  |  |  |  | **Inpatient** | | | | **Outpatient** | | | |
|  |  | Safety analysis set | | Incidence of TRAEs | | Safety analysis set | | Incidence of TRAEs | | Safety analysis set | | Incidence of TRAEs | |
|  |  | n | (%) | n | (%) | n | (%) | n | (%) | n | (%) | n | (%) |
| Number of patients | | 124 | (100.0) | 50 | (40.3) | 81 | (100.0) | 40 | (49.4) | 43 | (100.0) | 10 | (23.3) |
| Number of doses (times) | 1-4 | 35 | (28.2) | 15 | (42.9) | 27 | (33.3) | 13 | (48.1) | 8 | (18.6) | 2 | (25.0) |
|  | 5-8 | 36 | (29.0) | 15 | (41.7) | 20 | (24.7) | 10 | (50.0) | 16 | (37.2) | 5 | (31.3) |
|  | 9-12 | 35 | (28.2) | 14 | (40.0) | 22 | (27.2) | 12 | (54.5) | 13 | (30.2) | 2 | (15.4) |
|  | 13~ | 18 | (14.5) | 6 | (33.3) | 12 | (14.8) | 5 | (41.7) | 6 | (14.0) | 1 | (16.7) |

**Supplementary Table S4. Cross-tabulation of treatment-related adverse events (TRAEs; number of doses × ECOG Performance Status)**

| **Patient background factor** | | **Overall population** | | | | **ECOG Performance Status** | | | | | | | |
| --- | --- | --- | --- | --- | --- | --- | --- | --- | --- | --- | --- | --- | --- |
|  |  |  |  |  |  | **0-1** | | | | **2-4** | | | |
|  |  | Safety analysis set | | Incidence of TRAEs | | Safety analysis set | | Incidence of TRAEs | | Safety analysis set | | Incidence of TRAEs | |
|  |  | n | (%) | n | (%) | n | (%) | n | (%) | n | (%) | n | (%) |
| Number of patients | | 124 | (100.0) | 50 | (40.3) | 114 | (100.0) | 50 | (43.9) | 10 | (100.0) | 0 | - |
| Number of doses (times) | 1-4 | 35 | (28.2) | 15 | (42.9) | 27 | (23.7) | 15 | (55.6) | 8 | (80.0) | 0 | - |
|  | 5-8 | 36 | (29.0) | 15 | (41.7) | 35 | (30.7) | 15 | (42.9) | 1 | (10.0) | 0 | - |
|  | 9-12 | 35 | (28.2) | 14 | (40.0) | 34 | (29.8) | 14 | (41.2) | 1 | (10.0) | 0 | - |
|  | 13~ | 18 | (14.5) | 6 | (33.3) | 18 | (15.8) | 6 | (33.3) | 0 | - | 0 | - |

ECOG: Eastern Cooperative Oncology Group.

**Supplementary Table S5. Cross-tabulation of treatment-related adverse events (TRAEs; bodyweight × ECOG Performance Status)**

| **Patient background factor** | | **Overall population** | | | | **ECOG Performance Status** | | | | | | | |
| --- | --- | --- | --- | --- | --- | --- | --- | --- | --- | --- | --- | --- | --- |
|  |  |  |  |  |  | **0-1** | | | | **2-4** | | | |
|  |  | Safety analysis set | | Incidence of TRAEs | | Safety analysis set | | Incidence of TRAEs | | Safety analysis set | | Incidence of TRAEs | |
|  |  | n | (%) | n | (%) | n | (%) | n | (%) | n | (%) | n | (%) |
| Number of patients | | 124 | (100.0) | 50 | (40.3) | 114 | (100.0) | 50 | (43.9) | 10 | (100.0) | 0 | - |
| Body  weight (kg) | <50 | 24 | (19.4) | 7 | (29.2) | 20 | (17.5) | 7 | (35.0) | 4 | (40.0) | 0 | - |
|  | 50-60 | 48 | (38.7) | 16 | (33.3) | 43 | (37.7) | 16 | (37.2) | 5 | (50.0) | 0 | - |
|  | 60-70 | 36 | (29.0) | 19 | (52.8) | 36 | (31.6) | 19 | (52.8) | 0 | - | 0 | - |
|  | 70-80 | 11 | (8.9) | 5 | (45.5) | 10 | (8.8) | 5 | (50.0) | 1 | (10.0) | 0 | - |
|  | >80 | 4 | (3.2) | 3 | (75.0) | 4 | (3.5) | 3 | (75.0) | 0 | - | 0 | - |
|  | unknown | 1 | (0.8) | 0 | - | 1 | (0.9) | 0 | - | 0 | - | 0 | - |

ECOG: Eastern Cooperative Oncology Group.

**Supplementary Table S6. Cross-tabulation of treatment-related adverse events (TRAEs; case outcome × ECOG Performance Status)**

| **Patient background factor** | | **Overall population** | | | | **ECOG Performance Status** | | | | | | | |
| --- | --- | --- | --- | --- | --- | --- | --- | --- | --- | --- | --- | --- | --- |
|  |  |  |  |  |  | **0-1** | | | | **2-4** | | | |
|  |  | Safety analysis set | | Incidence of TRAEs | | Safety analysis set | | Incidence of TRAEs | | Safety analysis set | | Incidence of TRAEs | |
|  |  | n | (%) | n | (%) | n | (%) | n | (%) | n | (%) | n | (%) |
| Number of patients | | 124 | (100.0) | 50 | (40.3) | 114 | (100.0) | 50 | (43.9) | 10 | (100.0) | 0 | - |
| Case outcome | survival | 94 | (75.8) | 41 | (43.6) | 93 | (81.6) | 41 | (44.1) | 1 | (10.0) | 0 | - |
|  | death | 25 | (20.2) | 9 | (36.0) | 17 | (14.9) | 9 | (52.9) | 8 | (80.0) | 0 | - |
|  | unknown | 5 | (4.0) | 0 | - | 4 | (3.5) | 0 | - | 1 | (10.0) | 0 | - |

ECOG: Eastern Cooperative Oncology Group.
